# Soluble Guanylate Cyclase Stimulators in Heart Failure

**DOI:** 10.7759/cureus.17781

**Published:** 2021-09-06

**Authors:** Sajog Kansakar, Ashish Guragain, Deepak Verma, Prabhat Sharma, Bibek Dhungana, Bidit Bhattarai, Sunil Yadav, Nishant Gautam

**Affiliations:** 1 Internal Medicine, Manipal College of Medical Sciences, Pokhara, NPL; 2 Internal Medicine, Manipal College Of Medical Sciences, Pokhara, NPL; 3 Internal Medicine/Family Medicine, California Institute of Behavioral Neurosciences and Psychology, Fairfield, USA; 4 Internal Medicine, Shankarapur Hospital, Kathmandu, NPL; 5 Internal Medicine, KIST Medical College, Lalitpur, NPL; 6 Internal Medicine, Venus Hospital, Kathmandu, NPL; 7 Internal Medicine, Topa Primary Health Centre, Rajbiraj, NPL; 8 Internal Medicine, Bhaktapur Hospital, Bhaktapur, NPL

**Keywords:** heart failure with reduced ejection fraction (hfref), heart failure with preserved ejection fraction (hfpef), heart failure, guanylate cyclase stimulators, vericiguat

## Abstract

Heart failure has a high global burden of morbidity and mortality. Despite significant advances in medical management of heart failure, the prognosis remains poor. This justifies the search for newer therapeutic agents. Recently, soluble guanylate stimulators have demonstrated favorable results in clinical trials. This article aims to summarize the guanylate cyclase signaling pathway, the role of soluble guanylate cyclase stimulators in heart failure, and data from recent clinical trials of these drugs. We concluded that soluble guanylate cyclase stimulators have significant benefits in reducing hospitalizations in patients with heart failure with reduced ejection fraction that are at high risk of cardiovascular events. There appears to be no benefit of these drugs in patients with heart failure with preserved ejection fraction.

## Introduction and background

Heart failure is a clinical syndrome resulting from an impairment of ventricular filling or ejection of blood from the heart, which can manifest as dyspnea, exercise intolerance, and fluid retention [1]. There are several underlying causes of heart failure and often multiple causes co-exist to develop heart failure in a patient. These include ischemic heart disease, hypertension, valvular heart disease, genetic abnormalities, toxins, radiation, infections, infiltrative conditions, high output states, arrhythmias, etc. [2]. Heart failure can be classified based on left ventricular ejection fraction (LVEF) as follows: heart failure with reduced ejection fraction (HFrEF) with LVEF ≤ 40%, heart failure with mid-range ejection fraction (HFmrEF), or heart failure with borderline ejection fraction (HFbEF) with LVEF 41% to 49%, and heart failure with preserved ejection fraction (HFpEF) with LVEF ≥ 50% [1-2].

The diagnosis of heart failure is based on suggestive signs and symptoms (breathlessness, paroxysmal nocturnal dyspnea, orthopnea, fatigue, poor exercise tolerance, ankle swelling, elevated jugular venous pressure, laterally displaced apical impulse, third heart sound), prior clinical history (coronary artery disease, hypertension, toxins, radiation), resting electrocardiogram, natriuretic peptides, and echocardiogram [2-3]. The medical management of HFrEF includes primary treatment with angiotensin receptor-neprilysin inhibitor, angiotensin-converting enzyme inhibitor, or angiotensin receptor blocker, in combination with a beta blocker in all patients. Secondary agents, such as mineralocorticoid receptor antagonist, hydralazine plus nitrate, and ivabradine, can be provided in selected cases [4]. However, in terms of HFpEF and HFbEF, medications that improve mortality or clinical outcomes are yet to be determined.

Heart failure remains a global epidemic with an estimated prevalence of over 64 million worldwide [5]. In the United States (US), heart failure has a prevalence of over 6.5 million and accounts for one million cases of hospital admissions annually [6]. The incidence and prevalence demonstrate a growing trend, reflecting an aging population and rising prevalence of predisposing factors, such as obesity, diabetes, and hypertension. Despite significant advances in the pharmacological management of heart failure over the years, the prognosis remains poor with a five-year survival after hospitalization of 24.7% and a median survival of 2.1 years after admission [7]. Thus, the significant morbidity and mortality of heart failure warrant the search for newer therapeutic agents.

Soluble guanylate cyclase (sGC) stimulators have been in use for pulmonary arterial hypertension and chronic thromboembolic pulmonary hypertension but have recently emerged as promising agents for heart failure as well [8]. In this review, we aim to discuss the soluble guanylate cyclase signaling pathway, the role of soluble guanylate cyclase stimulators in heart failure, the data from recent clinical trials of these drugs, and their future implications.

## Review

The guanylate cyclase signaling pathway and mechanism of soluble guanylate cyclase stimulators

The nitric oxide (NO), soluble guanylate cyclase (sGC), cyclic guanosine 3,5-monophosphate (cGMP) signaling pathway plays a role in many processes of the human body, including a major role in the normal physiology of blood vessels [9]. Endogenous NO is synthesized in the vasculature from L-arginine by the enzyme endothelial NO synthase. Endothelial NO synthase can be activated via ligands, such as acetylcholine, adenosine, and bradykinin, as well as sheer stress on the endothelial cells. The NO that is produced in the endothelium travels to the smooth muscle layer of blood vessels where it binds to the heme group of the enzyme sGC and activates it. sGC is an enzyme that catalyzes the conversion of guanosine triphosphate to cGMP. cGMP mediates the downstream effects of this pathway, which includes vasodilation and subsequent hypotension, inhibition of platelet aggregation, inhibition of fibrosis, and inhibition of smooth muscle proliferation [10]. These downstream effects are mediated by the cGMP targets, such as cGMP-regulated protein kinases, that phosphorylate many types of molecules, cGMP-regulated phosphodiesterases, and cGMP-regulated ion channels [11]. Phosphodiesterases mediate the breakdown of cGMP, terminating the effects of this pathway [9].

In the heart, natriuretic peptides lead to the activation of the NO-sGC-cGMP pathway. Subsequent production of cGMP leads to activation of protein kinases that phosphorylate target proteins. This results in cardioprotective actions, such as natriuresis, improved diastolic relaxation, improved coronary blood flow, reduced hypertrophy, inflammation, and fibrosis [12].

**Figure 1:**
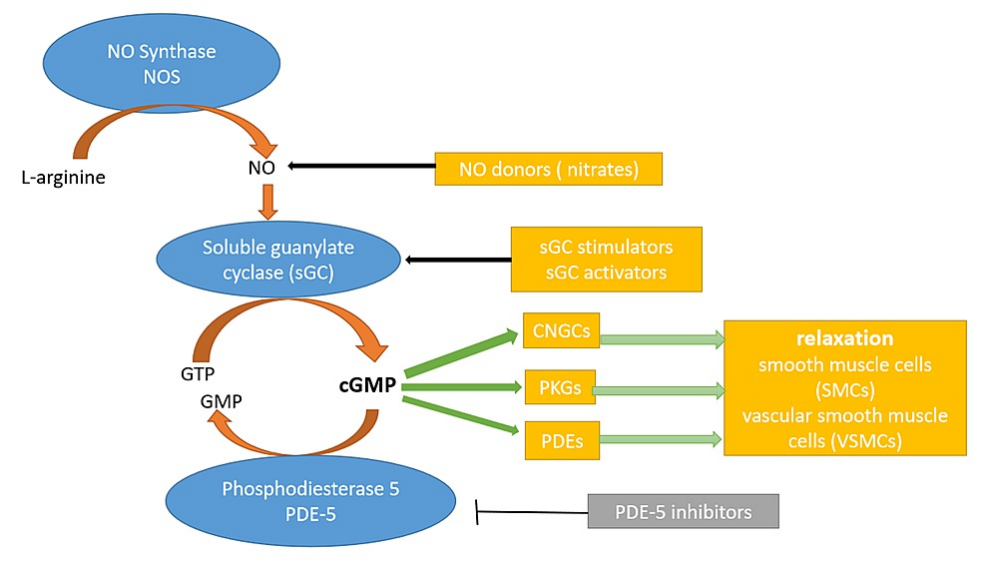
NO-sGC-cGMP pathway cGMP: cyclic guanosine monophosphate; CNGC: cyclic nucleoside gated-ion channels; GMP: guanosine monophosphate; GTP: guanosine triphosphate; NO: nitric oxide; NOS: nitric oxide synthase; PDE: phosphodiesterase; PKG: protein kinase G; sGC: soluble guanylate cyclase

sGC stimulators activate sGC independent of NO by binding to a non-heme site in sGC, which was unknown until recently [13]. A study by Wales et al. reports the binding site of sGC stimulators in the β1 heme nitric oxide/oxygen (H-NOX) domain of sGC [14]. Furthermore, sGC also augments the effects of NO by stabilizing the nitrosyl-heme complex that is formed after the binding of NO to the heme moiety of sGC, which keeps sGC in its active conformation for a longer period [15]. Figure 1 below depicts the NO-sGC-cGMP pathway.

The guanylate cyclase pathway in heart failure

The NO-sGC-cGMP signaling pathway in the heart has the above-mentioned actions, such as natriuresis, improved diastolic relaxation, coronary blood blow, and reduced hypertrophy, inflammation, and fibrosis. Thus, natriuretic peptides are an important mechanism opposing the effects of neurohormonal activation (renin-angiotensin-aldosterone system and sympathetic nervous system) that is seen in heart failure. However, in patients with heart failure, disruption of the NO-sGC-cGMP signaling pathway is seen. Comorbidities and hypoperfusion result in a state of oxidative stress and inflammation in the heart [12, 16]. The oxidative stress leads to reduced production of NO, excessive degradation of NO, and impaired responsiveness of sGC to endogenous NO, which leads to reduced functioning of the NO-sGC-cGMP signaling pathway [17]. Poor response to natriuretic peptides leads to worsening of the vicious cycle of neurohormonal activation in heart failure. Due to this disruption in the NO-sGC-cGMP signaling pathway, there is a rationale for the development of newer drugs in heart failure to target it. For example, the PARADIGM-HF (Prospective Comparison of ARNI with ACEI to Determine Impact on Global Mortality and Morbidity in Heart Failure) trial demonstrated that sacubitril-valsartan, a combination of neprilysin inhibitor and angiotensin receptor blocker, was superior to enalapril in reducing deaths and hospitalizations due to heart failure [18]. However, other drugs targeting the NO-sGC-cGMP signaling pathway, such as phosphodiesterase 3 inhibitor (milrinone, enoximone) and natriuretic peptide analogues (nesiritide), have not demonstrated significant benefits [19-21]. 

**Figure 2:**
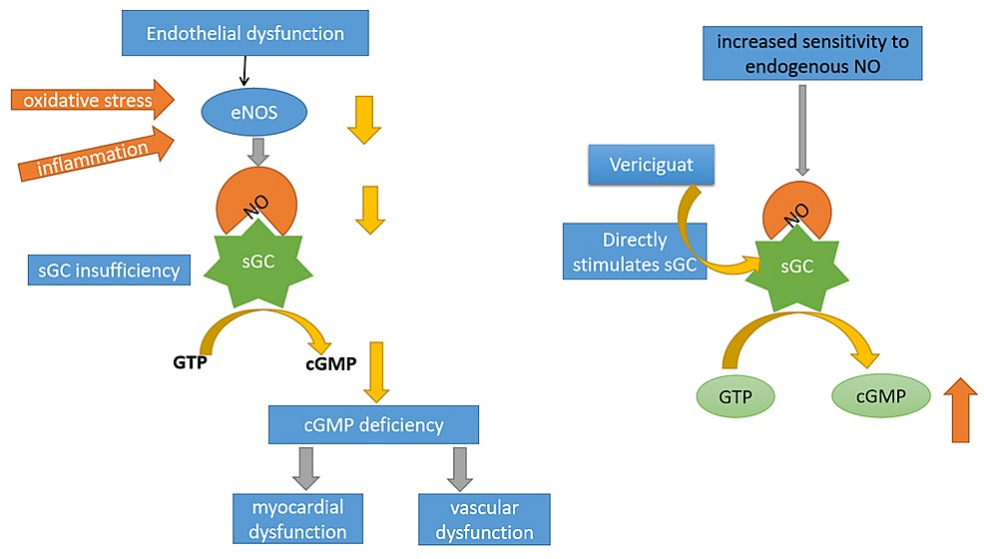
Mechanism of action of vericiguat cGMP: cyclic guanosine monophosphate; eNOS: endothelial nitric oxide synthase; GTP: guanosine triphosphate; NO: nitric oxide; sGC: soluble guanylate cyclase

Soluble guanylate stimulators work independent of NO and thus may be effective molecules to activate the NO-sGC-cGMP signaling pathway in a state of low NO which is seen in heart failure due to oxidative stress [16]. Figure 2 below depicts the mechanism of action of the soluble guanylate cyclase stimulator, vericiguat.

Soluble guanylate cyclase stimulators in clinical trials

The clinical utility of soluble guanylate stimulators were first observed with the results of the DILATE (Acute Hemodynamic Effects of Riociguat in Patients With Pulmonary Hypertension Associated With Diastolic Heart Failure) clinical trial [22]. The goal of the randomized, double-blinded study was to assess the safety and efficacy (in terms of hemodynamics) of riociguat (Adempas) in patients with pulmonary hypertension associated with HFpEF. The primary outcome was a change in mean pulmonary artery pressure from baseline after six hours and the secondary outcomes were other hemodynamic parameters, echocardiographic parameters, and safety. Thirty-nine patients were randomized to receive placebo or riociguat, 0.5 mg, 1 mg, or 2 mg in ascending doses. Although the primary outcome showed no significant difference between the groups, the riociguat 2 mg group showed a significant increase in stroke volume and cardiac index. It also decreased blood pressure and systemic vascular resistance. The adverse events were comparable between the groups, and it was concluded that riociguat demonstrated favourable effects in hemodynamic and echocardiographic parameters in patients with HFpEF and pulmonary hypertension.

The SOCRATES-REDUCED (Safety and Efficacy Study of Four Dose Regimens of BAY1021189 in Patients With Heart Failure With Reduced Ejection Fraction Suffering From Worsening Chronic Heart Failure) study was a Phase 2b double-blinded, clinical trial that included 456 patients with heart failure with an LVEF < 45% and a history of decompensation within the last four weeks [23]. The primary endpoint was the difference in NT-proBNP change in comparison to baseline. Patients were randomized to arms of placebo or oral vericiguat of either 1.25 mg, 2.5 mg, 5 mg, or 10 mg for 12 weeks. Seventy-seven percent of the patients completed the treatment. In the primary analysis, the difference in NT-proBNP was not statistically significant between placebo and pooled vericiguat groups. In a secondary analysis, however, higher doses of vericiguat resulted in a greater reduction in NT-proBNP. Between the placebo and vericiguat 10 mg group, the number of adverse events were similar.

Similarly, SOCRATES-PRESERVED (Safety and Efficacy Study of Four Dose Regimens of BAY1021189 in Patients With Heart Failure and Preserved Ejection Fraction Suffering From Worsening Chronic Heart Failure) was also a Phase 2b, double-blinded clinical trial with 477 patients with chronic heart failure with an LVEF > 45% and a history of decompensation within the last four weeks [24]. Patients were given a placebo or vericiguat, which was titrated from 1.25 mg to 10 mg once daily for 12 weeks. The change in NT-proBNP and left atrial volume in comparison to baseline were the primary outcomes. In contrast to SOCRATES-REDUCED, no statistically significant decrease in NT-proBNP was seen with vericiguat. However, improvement in quality of life scores were seen, which was measured by Kansas City Cardiomyopathy Questionnaire (KCCQ) and EuroQol 5 Dimension 5 Level (EQ-5D-5L).

Results of a larger Phase 2b clinical trial to assess the efficacy of vericiguat in patients with PHpEF were published in 2020. VITALITY-HFpEF (Patient-reported Outcomes in Vericiguat-treated Patients with HFpEF) was a randomized, double-blinded trial with 789 patients with chronic heart failure with LVEF > 45%, New York Heart Association Class II and III, a history of decompensation within the last six months, and elevated natriuretic peptides [25]. Patients were randomized equally to groups of placebo or vericiguat, 15 mg daily, or vericiguat, 10 mg daily, and followed up for 24 weeks. The primary outcome was a change in the physical limitation score of the KCCQ. At the end of 24 weeks, the differences among the three groups were not significant.

The results of the CAPACITY-HFpEF trial (A Study of the Effect of IW-1973 on the Exercise Capacity of Patients with Heart Failure with Preserved Ejection Fraction) were published in 2020 as well [26]. The goal of the trial was to assess the efficacy and safety of praliciguat in patients with HFpEF. One hundred and ninety-six patients with chronic heart failure and an LVEF > 40%, impaired peak oxygen consumption, and diabetes, hypertension, obesity, or advanced age were randomized equally into groups receiving placebo or praliciguat, 10 mg, 20 mg, or 40 mg, for 12 weeks. Eighty-six percent of participants completed the trial. The primary outcome was a change in peak oxygen consumption from baseline and secondary outcomes were a change in the six-minute walk test distance and ventilation efficiency (ventilation/carbon dioxide production slope) from baseline. For adverse effects, the primary outcome was the incidence of adverse effects emergence after treatment. None of the outcomes were significantly different among the various groups.

The results of the Phase 3 VICTORIA trial (Vericiguat Global Study in Subjects with Heart Failure with Reduced Ejection Fraction) were published in 2020 [27]. It was a double-blinded clinical trial including 5,050 patients with chronic heart failure (New York Heart Association Class II to IV) with an LVEF < 45%, history of decompensation over the last six months, elevated NT-proBNP or BNP. Participants received either vericiguat, 10 mg once daily, or a placebo. They were also given standard medical therapy directed by guidelines. The median follow-up was 10.8 months. The composite index of hospitalization for heart failure and death from cardiovascular causes was lower in the vericiguat group compared to placebo (35.5% vs 38.5%; hazard ratio: 0.90 [95% CI, 0.82 - 0.98]). However, in terms of death from cardiovascular causes only, no statistically significant difference was seen compared to placebo (16.4% vs 17.5%; hazard ratio: 0.93 [95% CI, 0.81 - 1.06]). The adverse effects incidence were not significantly different between the groups. Thus, vericiguat showed significant benefit in patients with HFrEF and was tolerable. However, the results of the VICTORIA trial cannot be generalized to populations with chronic heart failure without a high risk of cardiac events as the study only recruited patients with higher risk HFrEF. Table 1 below summarizes the major clinical trials involved in the study of the sGC stimulator, vericiguat.

**Table 1:** Summary of Clinical Trials BNP: brain natriuretic peptide; CAPACITY-HfpEF: A Study of the Effect of IW-1973 on the Exercise Capacity of Patients With Heart Failure With Preserved Ejection Fraction; DILATE: A Study to Test the Effects of Riociguat in Patients With Pulmonary Hypertension Associated With Left Ventricular Diastolic Dysfunction; HF: heart failure; HFpEF: heart failure with preserved ejection fraction; HFrEF: heart failure with reduced ejection fraction; HHF: hospitalization for heart failure; KCCQ: Kansas City Cardiomyopathy Questionnaire; LAV: left atrial volume; LVEF: left ventricular ejection fraction; mPAP: mean pulmonary arterial pressure; NCT: National Clinical Trial; NT-proBNP: N-terminal prohormone of brain natriuretic peptide; PH: pulmonary hypertension; PLS: physical limitation score; sGC: soluble guanylate cyclase; SOCRATES-PRESERVED: Phase IIb Safety and Efficacy Study of Four Dose Regimens of BAY1021189 in Patients With Heart Failure and Preserved Ejection Fraction Suffering From Worsening Chronic Heart Failure; SOCRATES-REDUCED: Phase IIb Safety and Efficacy Study of Four Dose Regimens of BAY1021189 in Patients With Heart Failure With Reduced Ejection Fraction Suffering From Worsening Chronic Heart Failure; VICTORIA: vericiguat Global Study in Subjects with Heart Failure with Reduced Ejection Fraction; VITALITY-HFpEF: Patient-reported Outcomes in Vericiguat-treated Patients With HFpEF

Author	Year	Clinical trial	Trial registration number	No. of patients	Purpose of study	Conclusions
Bonderman et al. [22]	2014	DILATE	NCT 01172756	39	To determine acute hemodynamic effects of riociguat in patients with PH and HFpEF	No significant difference in mPAP was seen between riociguat and placebo. However, riociguat increased the cardiac index.
Gheorghiade et al. [23]	2015	SOCRATES-REDUCED study	NCT 01951625	351	To determine optimal dose and tolerability of vericiguat in HFrEF patients	Vericiguat was well-tolerated and higher doses were associated with a greater reduction in NT-pro BNP level
Pieske et al. [24]	2017	SOCRATES-PRESERVED study	NCT 01951638	477	To determine the tolerability and optimal dose regimen of sGC stimulator, vericiguat in patients with HFpEF.	No significant change in NT-proBNP and LAV was seen in patients receiving vericiguat compared to placebo. Vericiguat was well-tolerated and associated with improved quality of life in patients with HFpEF.
Armstrong et al. [25]	2020	VITALITY-HFpEF study	NCT 03547583	789	To assess the efficacy of vericiguat on the PLS score of KCCQ	24-week treatment with vericiguat at either 15 mg/day or 10 mg/day did not improve the PLS of KCCQ compared to placebo.
Udelson et al. [26]	2020	CAPACITY-HFpEF	NCT 03254485	181	To assess the efficacy and safety of praliciguat on HFpEF patients.	No significant change in peak rate of oxygen consumption, six-minute walk test distance, and ventilator efficiency was seen. Praliciguat was well-tolerated.
Armstrong et al. [27]	2020	VICTORIA	NCT 02861534	5050	To assess the effect of vericiguat on death from cardiovascular causes or HHF in patients with HFrEF.	The composite index of hospitalization for heart failure and death from cardiovascular causes was lower in vericiguat group compared to placebo.

## Conclusions

The global burden and prognosis of heart failure remain poor. Nevertheless, the search for newer agents that improve clinical outcomes continues. With regards to HFpEF and HFbEF, the evidence for agents that improve mortality remains elusive as clinical trials do not support the use of soluble guanylate cyclase stimulators in HFpEF. However, encouraging developments have been observed recently in the management of HFrEF, with dapagliflozin and vericiguat showing some promise. Based on the studies in this review, the use of vericiguat did not show any mortality benefit, although a significant reduction in hospitalization was seen in patients with HFrEF. It was also well-tolerated in terms of adverse effects. Thus, vericiguat may have a place in the management of HFrEF as a secondary agent in patients with a high risk of cardiovascular events. Further studies are recommended to determine the place of vericiguat in the management of HFrEF.
